# Barbigerone prevents scopolamine-induced memory impairment in rats by inhibiting oxidative stress and acetylcholinesterase levels

**DOI:** 10.1098/rsos.230013

**Published:** 2023-04-12

**Authors:** Shareefa A. AlGhamdi, Fahad A. Al-Abbasi, Amira M. Alghamdi, Asma B. Omer, Obaid Afzal, Abdulmalik S. A. Altamimi, Abdulaziz Alamri, Sami I. Alzarea, Waleed Hassan Almalki, Imran Kazmi

**Affiliations:** ^1^ Department of Biochemistry, Faculty of Sciences, King Abdulaziz University, Jeddah 21589, Saudi Arabia; ^2^ Experimental Biochemistry Unit, King Fahd Medical Research Center, King Abdulaziz University, Jeddah 21589, Saudi Arabia; ^3^ Department of Basic Health Sciences, Foundation Year for the Health Colleges, Princess Nourah Bint Abdulrahman University, Riyadh 11671, Saudi Arabia; ^4^ Department of Pharmaceutical Chemistry, College of Pharmacy, Prince Sattam Bin Abdulaziz University, Al-Kharj 11942, Saudi Arabia; ^5^ Department of Biochemistry, College of Science, King Saud University, Riyadh 11451, Saudi Arabia; ^6^ Department of Pharmacology, College of Pharmacy, Jouf University, Aljouf, Sakaka 72341, Saudi Arabia; ^7^ Department of Pharmacology, College of Pharmacy, Umm Al-Qura University, Makkah 21955, Saudi Arabia

**Keywords:** dementia, flavonoids, isoflavone, neuroprotective

## Abstract

The current study was designed for the evaluation of barbigerone on memory loss. In this experimental study, 24 Wistar rats (*n* = 6) were used. Control rats and scopolamine (SCOP)-treated control group rats were orally administered with 3 ml of 0.5% sodium carboxymethyl cellulose (vehicle), whereas barbigerone was (10 and 20 mg kg^−1^) administered orally to the rats from the test group. During the 14-day treatment, control group rats were given 3 ml kg^−1^ day^−1^ saline, and all other groups were administered SCOP (1 mg kg^−1^ day^−1^, i.p.) 1 h after barbigerone p.o. treatment. The spontaneous alternation activities, learning capacities of a rat's memory were tested with Morris water maze and Y-maze. Reduced glutathione, malondialdehyde, acetylcholine esterase (AChE) and catalase (CAT) levels were measured in rat brain tissue as oxidative stress/antioxidant markers. Moreover, the levels of tumour necrosis factor, interleukin-6 (IL-6) and IL-1*β* were also estimated. Treatment with barbigerone in SCOP-administered rats dramatically reduced SCOP-induced neurobehavioural deficits, oxidative stress and neuroinflammatory markers, improved endogenous antioxidants, and restored AChE activity. By improving cholinergic function and reducing oxidative damage, barbigerone could mitigate the effects of SCOP-induced changes in the brain.

## 1. Introduction

Alzheimer's disease (AD) is a neurological condition with increased cholinergic system abnormalities and irreversible memory loss [1,2]. Currently, more than 46 million inhabitants globally suffer from AD, and by 2050, the diseased population is expected to rise to 131.5 million [3,4]. Because the condition is so incapacitating, it is a massive economic and social burden [1–3]. The condition is still incurable, and there is no way to stop it from progressing [2,5]. AD is associated with several genetic signs. These include the Apolipoprotein E gene, Presenilin 1 and 2 (PSEN1 and PSEN2) genes and the Amyloid precursor protein gene. Several other genes have been linked to an increased risk of developing AD, including CLU, PICALM and TREM2 [6,7]. Macroscopically, the brain tissue of patients with AD shows atrophy, or shrinkage, especially in the hippocampus and other parts of the temporal lobe. Microscopically, the brain tissue shows the presence of abnormal structures, such as beta-amyloid plaques. These plaques are composed of a protein fragment, which disrupts the normal functioning of neurons, leading to the progressive decline of cognitive abilities and memory [8]. However, the current therapeutic options largely treat symptoms, slowing the progression of AD [2,9,10]. N-methyl-D-aspartate blockers and acetylcholinesterase enzymes are the most popular treatments for the disorder [1–3]. Several side effects have been linked to these medications [1–3]. As a result, considerable therapeutic research into the memory-enhancing properties of natural compounds is currently ongoing [5]. Animal dementia models have been generated in a variety of ways, each with a different pathophysiology background [5,11]. Scopolamine (SCOP) inhibits muscarinic receptors by acting as a competitive antagonist [1,5]. It disrupts central cholinergic processes in rodents, impairing memory and learning [1,5]. This is analogous to how AD kills cortical cholinergic neurons and decreases central cholinergic function [1,5]. As an anticholinergic, SCOP stops acetylcholine (Ach) from binding to its receptors, resulting in high levels of Ach [12]. The damage to the hippocampal nerves causes memory loss and learning difficulties [5,12].

AD, among other neurological conditions with detrimental effects on learning and memory, is a good candidate for the SCOP-induced amnesia model [13–15]. Recent studies have shown that the use of SCOP causes cognitive dysfunction in rats and alteration in the oxidative stress of the brain [16,17]. Over-activity of reactive nitrogen species and reactive oxygen species (ROS) such as peroxynitrite-ONOO-, nitric oxide-NO, hydroxyl radicals-OH and superoxide anion radicals-O2-can result in oxidative stress that damages lipids, proteins, cell membranes and DNA. This can hasten the ageing and deformation of cells [18,19]. Anti-inflammatory and antioxidant medicines showed positive effects on a variety of CNS illnesses in recent studies [20–23]. In neurodegenerative illnesses, flavonoids showed anti-inflammatory and oxidant activity, and influenced gene expression [24–26]. There has been evidence that flavonoids can protect against neurodegenerative diseases in animal paradigms [27,28].

**Figure 1.**
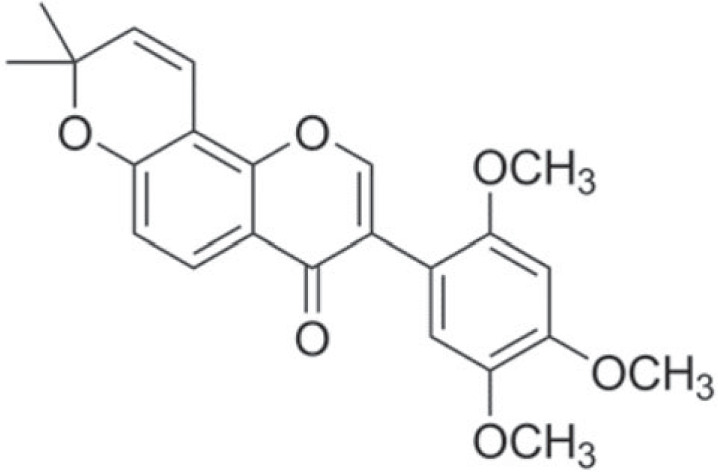
Chemical structure of barbigerone.

Isoflavones isolated from soya bean have been shown to be effective in amnesia in SCOP-treated mice [29]. Dietary isoflavones possess neuroprotective properties in cerebral ischaemia in experimental animal models [30]. Barbigerone (2′,4′,5′-trimethoxy-6″,6″-dimethyl-6″H-pyrano-[2″,3″:7,8]isoflavone) (figure 1) is an isoflavone discovered in the *Tephrosia barbigera* (Leguminosae) [31]. Its chemical structure can be described as a flavonoid nucleus with a prenyl side chain at the C-8 position. The main pharmacophoric groups in barbigerone are the flavonoid nucleus and the prenyl side chain. Barbigerone is also abundant in *M. pachycarpa* [32], *M. usaramensis* [33], *M. dielsiena* [34] and *M. ferruginea* [35] belonging to the family Leguminosae. *Sarcolobus globosus* (Apocynaceae), a medicinal herb, has also been used to isolate barbigerone [36]. Barbigerone is a compound with antiangiogenic [37], antioxidant [38] and anti-inflammatory actions. Barbigerone has a strong anti-plasmodial effect on the malaria parasite *Plasmodium falciparum* [39]. Barbigerone has been shown to have anti-cancer action in murine lung cancer cells by causing apoptosis [40].

The current study examined the role of barbigerone in SCOP-induced memory deficit in rats as well as its potential role in inhibiting oxidative stress and acetylcholinesterase activities.

## 2. Materials and methods

### 2.1. Chemicals

SCOP (6533-68-2) was obtained from Sigma-Aldrich. Barbigerone (98% purity, gift sample form SRL, India), and kits for interleukin-1*β* (IL-1*β*; MBS2023030), IL-6 (MBS2020158), and tumour necrosis factor (TNF-α; MBS9501941) were examined by rat enzyme-linked immune-sorbent examine kit (MyBioSource, USA). The experiment was carried out with high-grade quality chemicals and reagents.

### 2.2. Animals

Male Wistar rats (180 ± 20g) were acclimated to the working environment. Laboratory conditions were used, with 12 h of light and darkness each, and the animals had free access to food and water. The procedure was permitted by the Institutional Animal Ethics Committee (IAEC-TRS/PT/021/006-021) and adhered to CPCSEA rules.

### 2.3. Acute toxicity study

Barbigerone was tested for acute oral toxicity (LD50) by applying the OECD's-423 criteria. Barbigerone was dissolved in a 0.5% solution of sodium carboxymethyl cellulose (Na-CMC) and administered orally at the maximum dosage as reported previously [41,42].

### 2.4. Experimental design

A fresh solution of SCOP (1 mg kg^−1^) was given intraperitoneally (i. p.) in saline (pH 7.4) to induce memory impairment in rats [17,43]. A total number of 24 Wistar rats are classified into the following four groups;

Group 1: Control (Na-CMC-0.5% w/v, 3 ml kg^−1^)

Group 2: SCOP control groups (Na-CMC-0.5% w/v, 3 ml kg^−1^)

Group 3: SCOP + Barbigerone −10 mg kg^−1^, p.o. [40]

Group 4: SCOP + Barbigerone −20 mg kg^−1^, p.o. [40,42]

**Figure 2.**
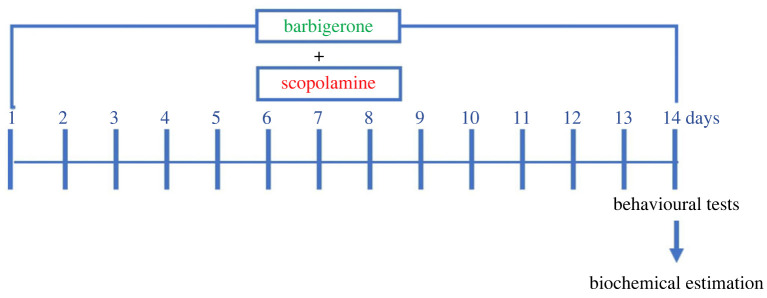
The schematic representation of the experimental procedure.

Barbigerone was administered orally 1 h before SCOP administration and at the end of 14 days 2 h after SCOP administration rats were exposed to animal behavioural tests, i.e. Morris water maze (MWM, days 14–18) and Y-maze test (days 14) [41,44,45]. Brains were extracted from animals for biochemical testing [17,43]. Figure 2 shows a schematic illustration of the experimental model.

### 2.5. Behavioural parameters

#### 2.5.1. Morris water maze paradigm test

Long-term memory impairment and spatial learning were determined using the MWM test. Aksoz *et al*.'s [46] methods were used to measure the MWM examination. The water in the tank was maintained at a constant temperature of 24 ± 10°C using creamy milk. The tank had four equally spaced quadrants. The platform was positioned in one quadrant and placed at 2.0 cm depth on the water surface. The platform was placed in the same quadrant throughout the experiment. On each day of the learning activity, one of the animals had been placed in the tank's randomly selected positions (three trials for each event). For the commencement of the experiment, the animals were brought further into the tank. As soon as the animals were found and stepped out onto the platform the experiment terminated, and mean escape latency was calculated. Sixty seconds was the highest exposed time. Another 60 s escape latency was observed if the animal failed to reach the platform even after initial pushing in 60 s. Following sessions, the animal was kept on the stand for 20 s. In all three trials, the animals were gently cleaned, followed by being placed in cages. On the fifth day of the consolidation trial, the rat's memory of the place of the hidden platform for 60 s was evaluated. The platform was pulled out from the tank during this stage. Thus, the time spent in the target quadrants where the hidden platform was placed had been noted.

#### 2.5.2. Y maze test

The Y-maze (45 × 12 × 35 cm) was composed of three identical arms that deviated at a 120° angle from one another, and a mid-symmetrical triangular area labelled A, B and C. During a 5 min session, the animal was positioned towards the end of one arm and had free access to movement throughout the maze. Animals must use working memory for spontaneous change, and the y-maze can be implemented for the assessment of spatial working memory. Therefore, they should trace the most recently visited arms, and this information should be renewed regularly. When the rat's hind paws were totally on the arm, the arm entry was considered complete. An alternate triplet set is characterized by successive entries into its three arms (i.e. ABC, BCA and CAB). Animals were evaluated for their memory loss as per the previously published protocol [47].

### 2.6. Biochemical parameters

#### 2.6.1. Tissue homogenization

Wash brain tissue was prepared in Isotonic saline (ice-cold), and phosphate buffer (0.1 M, pH 7.4, ice-cold) was used for the preparation of tissue homogenate whereas the biochemical analysis was performed on the supernatant [23,46,48,49].

#### 2.6.2. Acetylcholin esterase activity

Acetylcholin esterase (AChE) activity (µM mg^−1^ protein) was calculated as per the protocol published by Ellman *et al*. [50]. Briefly, homogenized aliquoted brain tissue was centrifuged at 14 000 × g for 5 min after being dissolved in 0.1 M (pH 7.5) PBS. The clear supernatants were separated to determine AChE activity and estimated using the kit as per the manufacturer's instructions. Briefly, a weighed amount of reagent was dissolved in the assay buffer to prepare the fresh working reagent.

In a 96-well plate, µl of sample and freshly prepared working reagent (190 µl) were combined by gently tapping the plate. As a reference, 200 µl of water (an assay blank) and 200 µl of calibrator were poured into separate wells. After incubating the samples at room temperature for about 2 min, the initial absorbance at 412 nm was measured. The plate was continuously incubated at room temperature, and the final absorbance was noted after 10 min of reaction. The activity of AChE was determined and measured as U mg^−1^ protein.

#### 2.6.3. Endogenous antioxidants

Reduced glutathione (GSH): GSH levels in the homogenates of brain tissue were measured following the method described by Ellman [51]. Proteins were precipitated by adding 100 µl (25%) TCA to 500 µl of homogenate, which was then centrifuged for 5 min at 4000 rpm, and the supernatant was collected. The supernatant (300 µl) was mixed with 500 µl of phosphate buffer (0.1 M, pH 7.4), and 200 µl of DTNB (10 mM) was added and incubated for 10 min. Absorbance was measured at a wavelength of 412 nm against a blank [36]. GSH activity was measured in U mg^−1^ protein.

Superoxide dismutase (SOD): SOD activity was assessed as per the methods given by Misra & Frodvich [52]. The activity of the SOD enzyme was carried out by a photochemical method that involved the addition of 1.5 ml of the supernatant to a reaction mixture comprising 100 mM Tris/HCl (pH 7.8), 75 mM NBT, 2 M riboflavin, 6 mM EDTA and 200 l of the reaction mixture. The detection of blue formazan was carried out by measuring the change in absorbance at 560 nm resulting from the formation of formazan. The SOD activity was quantified as U mg^−1^. SOD activity was measured as U mg^−1^ protein.

Catalase (CAT): in the cuvette, 0.1 ml of the supernatant was added to 1.9 ml of phosphate buffer (pH 7.0, 50 mM). Subsequently, the reaction was started by adding 1.0 ml of freshly made H2O2 (30 mM). CAT activity is calculated as U mg^−1^ protein [37].

Malondialdehyde: the level of malondialdehyde (MDA) in the homogenate of brain tissue was determined by the Wills method. After being placed in boiling water for 30 min, a test tube with 0.1 ml tissue homogenate, 1 ml TCA (10% w/v) and 1 ml TBA (0.67% w/v) was added. Subsequently, the test tubes were cooled in an ice bath for 10–15 min and centrifuged at 4000 × g for 10 min. After separating the clear, pink supernatant, the absorbance was measured at 532 nm [38].

#### 2.6.4. Pro-inflammatory cytokines

Immunoassay kits were used to measure IL-1*β*, IL-6 and TNF-α levels in the homogenate of brain tissue samples. Marker concentrations expressed in pg ml^−1^ protein were calculated using standard curves.

### 2.7. Statistical analysis

As a result of using GraphPad, all results are presented as means with standard errors of the means (s.e.m.). ANOVA was used for behavioural and biochemical parameters followed by Tukey's *post hoc* analysis. A two-way ANOVA followed by a Bonferroni *post hoc* test was used to evaluate the MWM test. The significance level for the study was set at *p* *<* 0.05.

## 3. Results

### 3.1. Acute oral toxicity studies

Barbigerone was tested for acute oral toxicity (LD50) as per the OECD's 423 criteria. During the 14-day timeframe, no mortality or adverse effects were recorded as shown in table 1. Based on safety data 10 and 20 mg kg^−1^ barbigerone doses were selected.

**Table 1.** Behavioural observations after treatment with barbigerone in acute oral toxicity.

observations	acute toxicity
eyes	normal
respiration	normal
skin and fur	normal
somatomotar activity	normal
convulsions	not seen
tremors	not seen
salivation	not seen
diarrhoea	not seen
lethargy	not seen
sleep	normal
coma	not seen
mortality	not seen

### 3.2. Behavioural parameters

#### 3.2.1. Morris water maze

In the MWM test, SCOP impaired rats' memory, as a greater ability to escape from swimming and step on the immovable platform was observed as a result of latency.

**Figure 3.**
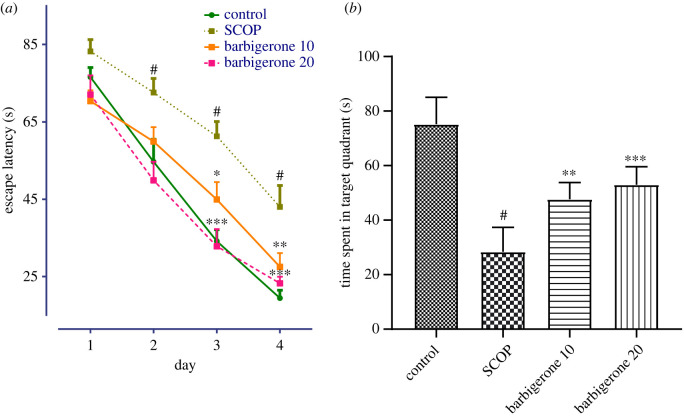
(*a,b*) Action of barbigerone on MWM test in rats treated with SCOP. (*a*) Escape latency, (*b*) time spent in target quadrants. ‘Mean ± s.e.m. (*n* = 6). #*p* *<* 0.0001 versus control, *p *<* 0.05, **p *<* 0.001 and ***p *<* 0.0001 versus SCOP control’.

The MWM test evaluated cognitive performance. During learning trials in all groups, the trained rats’ mean escape latency was reduced. SCOP-induced rats showed significantly increased latency time on the second, third and fourth days of training trials when compared to saline-control rats (*p* *<* 0.0001). Two-way ANOVA by a Bonferroni *post hoc* test showed that barbigerone administration significant reduced latency time in SCOP-treated rats (*p* *<* 0.0001). Additionally, the *post hoc* test showed that barbigerone administration (10 and 20 mg kg^−1^) significantly reduced latency time in comparison to SCOP-treated rats on days 2, 3 and 4 [*F*_3, 80_ = 108.0, (*p* *<* 0.0001)] (figure 3*a*).

The consolidation phase evaluates how effectively the rats retained and learned the hidden platform position during the 4 days of the trial. One-way ANOVA showed that in SCOP-treated rats less time was spent in the target quadrant in comparison to the saline-control rats, and barbigerone treatment significantly influenced the same (*p* *<* 0.0001). Additionally, the *post hoc* test showed that barbigerone (10 and 20 mg kg^−1^) enhanced time spent in the target quadrant in comparison to SCOP-treated rats [*F*_3, 20_ = 34.26, (*p* *<* 0.0001)] (figure 3*b*).

#### 3.2.2. Y-maze test

**Figure 4.**
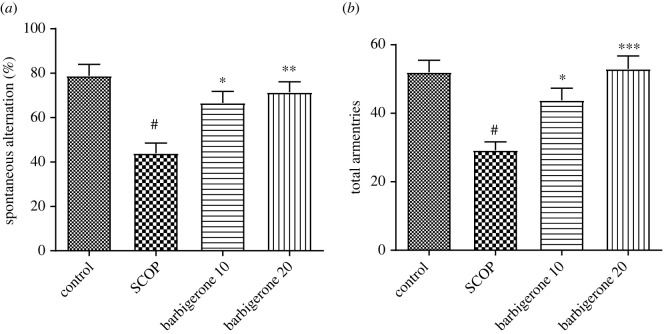
(*a,b*) Action of barbigerone on Y-maze test on rats treated with SCOP. (*a*) Spontaneous alternation and (*b*) total number of arm entries. ‘Mean ± s.e.m. (*n* = 6). ^#^*p* *<* 0.0001 versus control, *p *<* 0.05, **p *<* 0.001 and ***p *<* 0.0001 versus SCOP control’.

Spontaneous change and total arm entries were lower in SCOP-treated animals compared to control animals. The Y-maze test results were statistically substantial (*p* *<* 0.0001). Barbigerone-10 and 20 mg kg^−1^ administration to SCOP-administered rats increased total arm entries [*F*_4, 25_ = 8.948, (*p* *<* 0.0001)] and spontaneous alternation [*F*_3, 20_ = 9.441, (*p* = 0.0004)] versus SCOP control. Figure 4 represents the results of the Y-maze test (figure 4*a,b*).

### 3.3. Biochemical parameters

#### 3.3.1. Acetylcholin esterase activity

**Figure 5.**
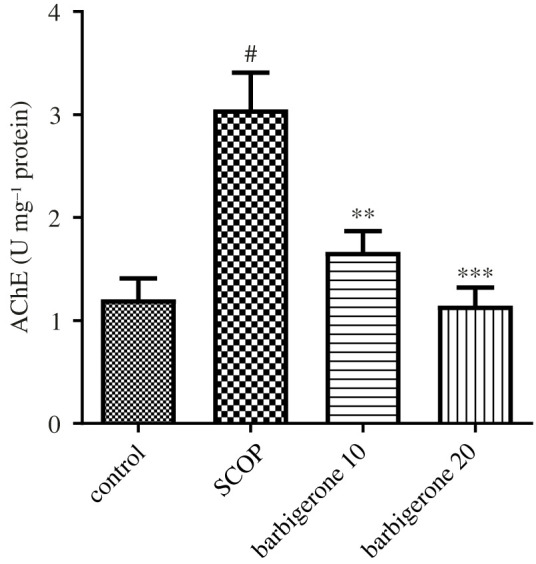
Action of barbigerone on AChE activity in rats treated with SCOP. ‘Mean ± s.e.m. (*n* = 6). ^#^*p* *<* 0.0001 versus control, **p *<* 0.001 and ***p *<* 0.0001 versus SCOP control’.

The SCOP control group had more than twofold higher AchE levels (*p* *<* 0.0001) than the control rats. Barbigerone (10 and 20 mg kg^−1^) significantly lowered AchE levels [*F*_3, 20_ = 12.99, (*p* *<* 0.0001)] in SCOP-treated rats versus SCOPcontrol animals. (figure 5).

#### 3.3.2. Endogenous antioxidants status

**Figure 6.**
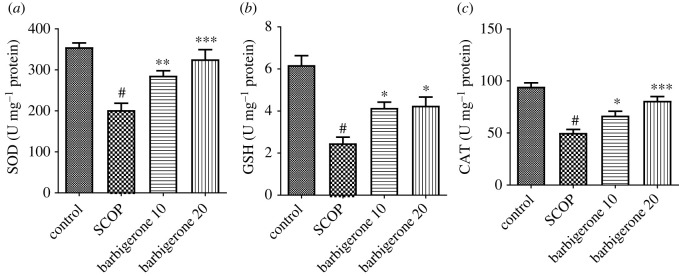
(*a–c*) Action of barbigerone on (*a*) SOD, (*b*) reduced GSH and (*c*) CAT in rats treated with SCOP. ‘Mean ± s.e.m. (*n* = 6). ^#^*p* *<* 0.0001 versus control, *p *<* 0.05, **p *<* 0.001 and ***p *<* 0.0001 versus SCOP control’.

The number of antioxidants (GSH, SOD and CAT) was reduced in SCOP-administered groups. The SCOP control group had considerably (*p* *<* 0.0001) less SOD, GSH and CAT than the control rats. Barbigerone (10 and 20 mg kg^−1^) effectively restored the level of SOD [*F*_3, 20_ = 16.12, (*p* *<* 0.0001)], GSH [*F*_3, 20_ = 17.33, (*p* *<* 0.0001)] and CAT [*F*_3, 20_ = 24.30, (*p* *<* 0.0001)] levels when compared to the SCOP control group. Figure 6*a–c* depicts the results of the endogenous antioxidant status test.

**Figure 7.**
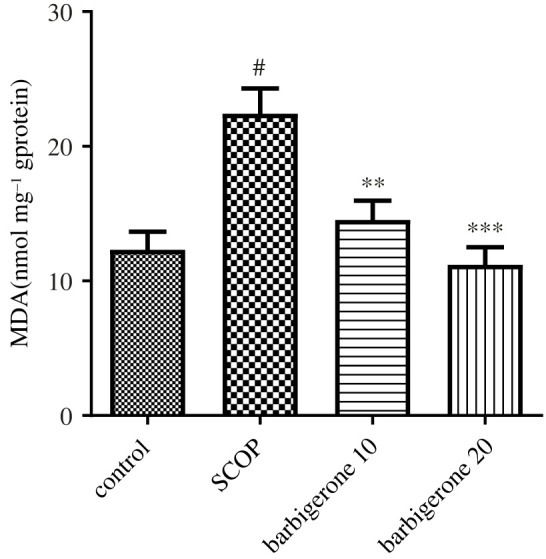
Effect of barbigerone on MDA levels in rats treated with SCOP. ‘Mean ± s.e.m. (*n* = 6). ^#^*p* *<* 0.0001 versus control, **p *<* 0.001 and ***p *<* 0.0001 versus SCOP control’.

When SCOP control animals were increased MDA levels were compared with control rats (*p* *<* 0.05). The MDA level significantly increased in the SCOP control group as compared to the control rats. Barbigerone (10 and 20 mg kg^−1^) administration significantly decreased MDA levels [*F*_3, 20_ = 11.42, (*p* *<* 0.0001)] when compared to SCOP-injected animals; figure 7 shows the levels of MDA level.

#### 3.3.3. Pro-inflammatory cytokines

**Figure 8.**
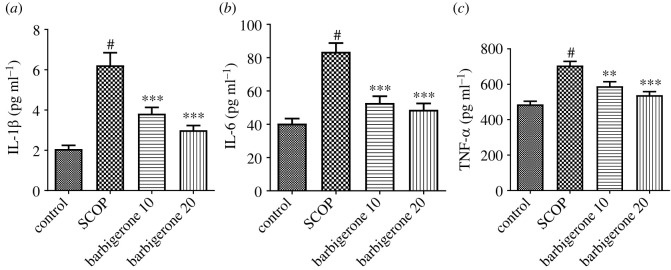
(*a–c*) Effect of barbigerone on pro-inflammatory cytokines in rats treated with SCOP. (*a*) IL-1*β*, (*b*) interleukin-6 (IL-6) and (*c*) TNF-α. ‘Mean ± s.e.m. (*n* = 6). ^#^*p* *<* 0.0001 versus control, **p *<* 0.001 and ***p *<* 0.0001 versus SCOP control’.

The IL-1*β*, IL-6 and TNF-α were significantly (*p* *<* 0.05) elevated in SCOP-treated rats in comparison to control rats. Administration of barbigerone (10 and 20 mg kg^−1^) significantly reduced IL-1*β* [*F*_3, 20_ = 22.26, (*p* *<* 0.0001)], IL-6 [*F*_3, 20_ = 21.05, (*p* *<* 0.0001)] and TNF-α [*F*_3, 20_ = 19.06, (*p* *<* 0.0001)] levels as compared to SCOP control rats. Figure 8*a–c* shows the findings of IL-1*β*, IL-6 and TNF-α tests.

## 4. Discussion

The effect of barbigerone on memory impairment induced by SCOP was studied using exteroceptive behavioural tasks, including the MWM and Y-maze tests. These paradigms have mostly been used for assessing animal learning and memory, most notably rodent behavioural influences [39].

In animal models of neurodegenerative illnesses, SCOP-induced memory impairment is extensively used to examine the causes and therapies of cognitive impairment [53].

Working memory is evaluated using the total arm entries and spontaneous change score in the Y-maze test [39]. In this study, total arm entries and spontaneous change decreased more in the SCOP group than in the control group. This shows that SCOP-treated animals have poor working memory. Barbigerone administration to SCOP-treated rats enhanced total arm entries and spontaneous change. These findings suggest that barbigerone protects treated rats against memory impairment caused by SCOP. Learning ability and long-term spatial memory can be assessed by the MWM test [1,2,12]. SCOP treatment delayed the escape latency indicating decreased learning ability and spatial memory in rats as well as reduced time spent in the target quadrant on the fifth day of the trial. Barbigerone treatment to SCOP-treated animals improved escape latency in the MWM paradigm. These results point to barbigerone's ability to protect animals from SCOP-induced memory deficits.

An ACh receptor antagonist, SCOP interrupts cholinergic neurotransmission and can cause learning and memory issues [45,54]. Learning and memory are both aided by the central cholinergic system [1], and ACh is a key neurotransmitter that modulates intellectual performance and learning processes [1,55]. Enzyme AChE metabolizes ACh in the synaptic cleft to acetic acid and choline [56]. On the other hand, AChE activity might lead to a lack of ACh and cognitive deficit [57]. In the current study, SCOP-induced animals improved AChE levels compared to control rats. However, barbigerone treatment with 10 and 20 mg doses to SCOP-treated animals significantly decreased AChE activity. These findings suggest that barbigerone can protect treated rats from SCOP-induced AChE activity.

Along with cognitive function, ACh is a key component of the cholinergic anti-inflammatory system [58]. Cholinergic anti-inflammatory mechanisms regulate IL-6, IL-1*β* and TNF-α, which promote inflammation and facilitate TNF-α separation, resulting in an inflammatory response. It also triggers and causes the death of nerve cells and synapses that contribute to neurodegeneration [58,59]. SCOP treatment increased IL-1*β*, IL-6 and TNF-α levels in the treated animals. Barbigerone therapy of SCOP-treated rats restored IL-6, TNF-α and IL-1*β* levels. The cytokines are proteins that are involved in the regulation of inflammation and the immune system, and when they are increased due to SCOP, this suggests that inflammation occurred. Barbigerone helped to restore the balance of the immune system by reducing the production of these cytokines, i.e. IL-6, TNF-α and IL-1*β* levels.

Along with the cholinergic theory, oxidative stress has been considered a probable cause of neurocognitive illness [60]. SCOP dramatically exacerbated oxidative stress in the rats, as demonstrated by greater MDA levels and lower levels of CAT, GSH and SOD [61,62]. Oxidative stress-driven illness occurs when levels of peroxides and ROS surpassed endogenous antioxidant defences [20,22]. In the brain, polyunsaturated fatty acids are damaged by lipid peroxidation [21,49]. Furthermore, the brain is especially prone to oxidative damage due to its lack of antioxidant defence mechanisms [21,49]. The use of barbigerone restores antioxidants and reduces MDA levels. Barbigerone's antioxidant properties may account for its neuroprotective effects in rats suffering from SCOP-induced neurotoxicity. These findings also suggest that barbigerone may have neuroprotective effects on the brain by reducing oxidative stress and improving cholinergic function. The limitations of the study are of short duration with the use of minimum animals. Based on the available evidence, this is the first study to show that barbigerone can protect against SCOP-induced memory loss in rats. This mechanism needs to be confirmed by further studies including immunohistochemistry and molecular studies, including western blotting, RT-PCR, tissue immunohistochemistry also with other model amyloid oligomers injections, streptozotocin and other genetic modes.

## 5. Conclusion

During the 14-days treatment schedule we observed that barbigerone could mitigate these SCOP-induced alterations in the brains of SCOP-treated rats by improving cholinergic function and decreasing oxidative damage. The current investigation reveals that SCOP-induced behavioural and physiological alterations in rats are reduced by the barbigerone, a flavonoid mainly lowering levels of the inflammatory response. However, more studies need to be performed to determine the impact of barbigerone on persons with memory problems.

## Ethical

The protocol used in this study was approved by the TRS, India-Institutional Animal Ethics Committee -IAEC-TRS/PT/021/006-021, as per CPCSEA and were reported in accordance with ARRIVE (Animal Research: Reporting of *In Vivo* Experiments) requirements [63].

Institutional Animal Ethics Committee (IAEC/TRS/PT/021/006-021).

## Data Availability

The datasets supporting this article have been uploaded as part of the electronic supplementary material https://10.6084/m9.figshare.21961622 [64]. The data are provided in the electronic supplementary material [65].
